# RNA-sequencing-based transcriptome and biochemical analyses of steroidal saponin pathway in a complete set of *Allium fistulosum*—*A*. *cepa* monosomic addition lines

**DOI:** 10.1371/journal.pone.0181784

**Published:** 2017-08-11

**Authors:** Mostafa Abdelrahman, Magdi El-Sayed, Shusei Sato, Hideki Hirakawa, Shin-ichi Ito, Keisuke Tanaka, Yoko Mine, Nobuo Sugiyama, Minoru Suzuki, Naoki Yamauchi, Masayoshi Shigyo

**Affiliations:** 1 Laboratory of Vegetable Crop Science, College of Agriculture, Graduate School of Sciences and Technology for Innovation, Japan; 2 Botany Department, Faculty of Science, Aswan University, Aswan, Egypt; 3 Graduate School of Life Sciences, Tohoku University, Aoba-ku, Sendai, Japan; 4 Kazusa DNA Research Institute, Kisarazu, Chiba, Japan; 5 Laboratory of Molecular Plant Pathology, College of Agriculture, Graduate School of Sciences and Technology for Innovation, Japan; 6 The NODAI Genome Research Center, Tokyo University of Agriculture, Tokyo, Japan; 7 Department of Agriculture, Faculty of Agriculture, Tokyo University of Agriculture, Tokyo, Japan; 8 Department of Computational Biology and Medical Sciences, Graduate School of Frontier Sciences, The University of Tokyo, Chiba, Japan; Youngstown State University, UNITED STATES

## Abstract

The genus *Allium* is a rich source of steroidal saponins, and its medicinal properties have been attributed to these bioactive compounds. The saponin compounds with diverse structures play a pivotal role in *Allium’s* defense mechanism. Despite numerous studies on the occurrence and chemical structure of steroidal saponins, their biosynthetic pathway in *Allium* species is poorly understood. The monosomic addition lines (MALs) of the Japanese bunching onion (*A*. *fistulosum*, FF) with an extra chromosome from the shallot (*A*. *cepa* Aggregatum group, AA) are powerful genetic resources that enable us to understand many physiological traits of *Allium*. In the present study, we were able to isolate and identify Alliospiroside A saponin compound in *A*. *fistulosum* with extra chromosome 2A from shallot (FF2A) and its role in the defense mechanism against *Fusarium* pathogens. Furthermore, to gain molecular insight into the *Allium* saponin biosynthesis pathway, high-throughput RNA-Seq of the root, bulb, and leaf of AA, MALs, and FF was carried out using Illumina's HiSeq 2500 platform. An open access *Allium* Transcript Database (*Allium* TDB, http://alliumtdb.kazusa.or.jp) was generated based on RNA-Seq data. The resulting assembled transcripts were functionally annotated, revealing 50 unigenes involved in saponin biosynthesis. Differential gene expression (DGE) analyses of AA and MALs as compared with FF (as a control) revealed a strong up-regulation of the saponin downstream pathway, including cytochrome P450, *glycosyltransferase*, and *beta-glucosidase* in chromosome 2A. An understanding of the saponin compounds and biosynthesis-related genes would facilitate the development of plants with unique saponin content and, subsequently, improved disease resistance.

## Introduction

*Allium* is an enormous genus (850 species) that stretches broadly across the northern hemisphere from the boreal zone to the dry subtropics [1–3]. A region with diverse ecological niches led to the development of an astonishing number of *Allium* species with different morphological and physiological traits [1]. Due to their culinary and medicinal properties, many plants of this genus [*A*. *cepa* (onion), *A*. *cepa* Aggregatum group (shallot), *A*. *fistulosum* (Japanese bunching onion), *A*. *sativum* (garlic), *A*. *ampeloprasum* (leek), and *A*. *tuberosum* (Chinese chives)] have significant economic importance worldwide as vegetables or medicinal plants [4–6]. However, genetic shifts and drastic unbalance selection by farmers and breeders have caused the loss of many useful agronomic traits in *Allium* [1]. Therefore, to develop disease-resistant *Allium* germplasm, novel alleles with desirable physiological attributes can be introduced by crossing with disease-resistant cultivars or wild relatives [1, 3, 4, 7].

The shallot is a species of subtropical origin that has been recognized as a potential genetic resource for *Allium* crop improvements because of their adaptability to environmental stresses [8, 9]. However, the molecular and physiological architecture underlying this tolerability is still unclear. The utilization of monosomic addition lines (MALs) as valuable genetic resources for understanding physiological traits has been reported in several plant species, including the *Beta vulgaris* L. genome with the addition of chromosome 9 from *B*. *corolliflora* to improve salt stress [10, 11] and the *Brassica napus* genome mediated by one alien chromosome from *Orychophragmus violaceus* for understanding the metabolism pathways regulating brassinosteroid (BR) biosynthesis and the role of auxin signaling in gynoecium development [12]. Our previous studies have revealed the significance of utilizing a shallot (AA) chromosomal engineering technique to improve *A*. *fistulosum* (FF) physiological traits that exhibited interesting phenotypes [13, 14]. MALs of *A*. *fistulosum* with an extra chromosome from the shallot enhanced the flavonoid [15], carbohydrate [16], cysteine sulfoxide [17] and saponin [18] contents. The increased saponin content in FF2A line was positively correlated with increased *Fusarium* disease resistance index [18]. These findings give insight into the significant role of shallot saponin in the disease resistance improvement of *A*. *fistulosum* despite the fact that the casual genes regulating this biosynthesis process in *Allium* are still unknown.

Steroidal saponins are synthesized via the mevalonic acid (MVA) pathway in the cytoplasm [19] and/or through the 2-C-methyl-D-erythritol 4-phosphate (MEP) pathway in plastids [20]. The cyclization of precursor compound 2, 3-oxidosqualene by *oxidosqualene cyclase* (*OSC*) combined with steroidal skeleton modifications through hydroxylation and glycosylation leads to the formation of various saponin compounds [21]. Several *OSC*-related genes, such as *cycloartenol synthase* (*CAS*), *lupeol synthase* (*LS*), and *beta-amyrin synthase* (*β-AS*), have been isolated from various plant systems [22–24]. According to the proposed pathway, some specific cytochrome P450, UDP-glucosyltransferases (UGT), and beta-glucosidase protein encoding genes are involved in the cyclization of the downstream pathway of the saponin biosynthesis [21, 25–27]. Despite many studies on the chemical structure and pharmaceutical activities of steroidal saponins, little is known about the molecular mechanism of the cyclization process involved at the downstream level.

For large-scale transcriptome analysis, next-generation sequencing (NGS) has rapidly evolved into an expedient technique for providing huge expression data in a much shorter time and has accelerated our understanding of metabolic pathways as well as contributing to gene discovery [9, 26, 28]. Transcriptome analysis, followed by the identification of prospective candidate genes involved in the secondary metabolic pathway, will lead to further understanding of biosynthesis and the diversity of secondary metabolites [29]. In the present study, we have performed phytochemical analyses to identify the shallot-specific saponin compound detected in FF2A, using column chromatography and two-dimensional nuclear magnetic resonance (2D NMR) spectroscopy. Phytochemical analyses resulted in the isolation and identification of a spirostanol saponin compound named Alliospiroside A. The antifungal activity of Alliospiroside A, the furostanol saponin fraction, and the root crude saponin extracts of AA, MALs (MALs = FF1A, FF2A, FF3A, FF4A, FF5A, FF6A, FF7A, and FF8A), and FF was examined against different *Fusarium* pathogens. Furthermore, to identify the candidate genes involved in saponin biosynthesis, high-throughput transcriptome analyses of the root, bulb, and leaf of AA, MALs, and FF were performed using NGS technology based on Illumina’s HiSeq 2500 platform. The resulting assembled transcripts were functionally annotated and used for DGE analyses of AA and MALs compared with FF (as a control). The DGE data were further used for saponin pathway analyses. The RNA-Seq data set including contigs length, nucleotide sequences, amino acid sequences, annotation, and expression values has been submitted to the open access *Allium* Transcript Database (*Allium* TDB, http://alliumtdb.kazusa.or.jp). Our ultimate goal is to discover the candidate genes that encode enzymes in the steroidal saponin biosynthetic pathway and to provide an overview of transcriptome dynamics in the different tissues as well as the role of shallot saponin in the defense mechanism against *Fusarium* pathogens. Our transcriptome dataset is a valuable and unique resource that will facilitate future functional genetics studies and molecular marker development for *Allium* breeding.

## Materials and methods

### Plant materials

*Allium cepa* Aggregatum group, monosomic lines, and *A*. *fistulosum* were grown in clay pots filled with sand (one plant per pot) under the same conditions at the Yamaguchi University greenhouse. The average temperature was 20 ± 2°C, relative humidity 78% and 10 h daylight length. Water and fertilizers were applied equally for all genotypes on weekly base. Plants were collected from each line separately in biological replica (n = 3). After cleaning, the root, bulb, and leaf [3 replicates × 3 tissues × 10 genotypes (AA, eight MALs, and FF)] were cut separately and immediately frozen in liquid nitrogen for RNA extraction and phytochemical analyses.

### AA, MALs, and FF saponin profiling

The extraction of the saponin from AA, MALs, and FF root was carried out in accordance with the procedures of Mostafa et al. [4]. Freeze-dried root (2 g) was exhaustively extracted at room temperature with the following solvents: 20 ml of *n*-hexane and 50 ml of 70% MeOH. Each solvent extraction step was conducted for one day and was repeated three times with 30 min of sonication and filtration. The MeOH extract was dried in a rotary evaporator with a vacuum pump (v-700; BUCHI, Rotavapor R-3, BÜCHI Labortechnik AG Postfach, Switzerland) under reduced pressure at 50°C and then partitioned between *n*-Butanol (BuOH) and H_2_O (1:1, v/v). The BuOH layer was filtered and concentrated under vacuum to afford saponin crude extracts. A small amount of the extracts (10–20 μl) were chromatographed using thin-layer chromatography (TLC) silica gel plates (60 F_254_; Merck KgaA, Darmstadt, Germany). The chromatogram was developed with CHCl_3_:MeOH:H_2_O (30:15:2.5, v/v/v). The plates were dried, and the spots were visualized using a p-anisaldehyde reagent for total saponins and Ehrlich's reagent for furostanol saponins.

### Determination of total saponin contents in the root, bulb and leaf of AA, MALs and FF

The extraction of the saponin from AA, MALs, and FF root, bulb and leaf was carried out as described above [4]. Total saponin contents were determined spectrophotometrically at 473 nm [3]. Saponin concentrations were calculated based on disogenin standard. All chemical extractions consisted of three replications.

### Extraction and isolation of shallot-specific saponin compounds

The extraction of saponins from FF2A root (25 g) was carried out as mentioned above. Aliquot of the crude extract was chromatographed by C300 silica gel column chromatography (3 cm × 60 cm; AG Tokyo, Japan). The column was developed using a gradient solvent system, starting with CHCl_3_, CHCl_3_:MeOH (9:1–1:9), MeOH, and MeOH:H_2_O (9:1–7:4) as eluents to give 8 fractions after the evaporation of solvents (F1–F8). Each fraction was rechromatographed using TLC silica gel plates. The chromatogram was developed with CHCl_3_:MeOH:H_2_O (30:15:2.5, v/v/v). Fraction 2 yielded a 10 mg pure compound that was subjected to 2D NMR analysis.

### 2D NMR spectroscopy

The structure of isolated saponin compound was elucidated using 2D NMR. Optical rotations were determined with the JASCO DIP-1000 digital polarimeter. ^13^C and ^1^H NMR spectra were recorded in a pyridine-*d5* solution at 500 and 125 MHz, respectively, using the JEOL ECA 500 spectrometer. The *J* values were expressed in hertz, and chemical shifts (δ) in parts per million (ppm), using pyridine-*d5* for ^13^C NMR (123.5 ppm) and ^1^H NMR (7.20 ppm). The high-resolution electrospray ionization mass spectrometry was recorded using the JEOL JMS-T100LP spectrometer.

### Biological assays

The antifungal activity of saponin extracts from AA, MALs, and FF root was examined against *F*. *oxysporum* f. sp. *cepa* strains TA and AF22. Pathogens were obtained from the Laboratory of Plant Molecular Pathology, Yamaguchi University, Japan. Antifungal activity was evaluated with the agar plate diffusion method, using 3.2-cm diameter Perspex plates with potato dextrose agar (PDA). Crude saponins were added to obtain the final concentration of 1000 μg ml^-1^, and the plates were inoculated with a 5-mm plug that contained the fungi grown on PDA for 5 days. The plates were incubated at 25 ± 2°C and the fungal radical growth was measured after one week. The antifungal activity of the furostanol saponin fraction and Alliospiroside A was examined against the respective strains as mentioned above at final concentration of 200 μg ml^-1^. All experiments were conducted in three replicates (n = 3).

### Construction of unigenes and functional annotation

Unigene sets were constructed by assembling the Illumina RNA-Seq sampled from the bulb of AA. The cDNA library was prepared in accordance with Illumina's protocol, and sequencing was performed using Illumina’s HiSeq 2500 platform. The reads including adapter sequence and unknown nucleotides more than 5% and low-quality nucleotides (QV≤10) more than 20% in length were respectively excluded. The remained reads were assembled into contigs by Trinity r20121005 [30] with parameters,—seqType fq—min_contig_length 100—group_pairs_distance 250—path_reinforcement_distance 85—min_kmer_cov 2. In each sample, the contigs were clustered by TGI Clustering Tool (TGICL) v2.1 [31] with parameters, -l 40 -c 10 -v 20, and further assembled into unigenes by Phrap 23.0 [32, 33] with parameters, -repeat stringency 0.95 -minmatch 35 -minscore 35. A total of 56,161 obtained unigene sequences were searched against the databases, the *Arabidopsis* Information Resource (TAIR10; http://www.arabidopsis.org), RAP-DB (International Rice Genome Sequencing Project (IRGSP-1.0; http://rgp.dna.affrc.go.jp/IRGSP/), and the NCBI’s non-redundant proteins (nr) database (http://www.ncbi.nlm.nih.gov), using BLASTX [34] program with an E-value cutoff of 1E-10. The functional categories of these unigene sets were assigned using Gene Ontology (GO) database (http://geneontology.org).

### RNA sequencing and read mapping of AA, MALs and FF

Total RNA was isolated using RNeasy Plant Mini Kit (QIAGEN Sciences, Germantown, Maryland, USA). Total RNA was assessed using BioSpec-nano (Shimadzu, Kyoto, Japan), and an additional RNA quality check was completed using the Agilent 2100 Bioanalyzer (Agilent Technologies, Palo Alto, CA, USA). Samples with RNA integrity number (RIN) values of more than 8.0 were selected for further use. The cDNA library was generated using TruSeq^™^ RNA Sample Preparation Kit (Illumina, San Diego, CA, USA) in accordance with the manufacturer's instructions. Briefly, oligo (dT) beads were used to purify poly (A) mRNA from total RNA. mRNA was fragmented using an RNA fragmentation kit (Ambion Life Technologies, USA). First-strand cDNA was synthesized from the fragmented mRNA using random hexamer primers and reverse transcriptase (Invitrogen Life Technologies, Carlsbad, CA, USA). The cDNA library was prepared in accordance with Illumina's protocol, and sequencing was performed using Illumina’s HiSeq 2500 platform. The trimmed RNA-Seq reads of each sample (AA, MALs, and FF) were respectively mapped onto the unigene sequences by Bowtie 2 ver. 2.2.0 with the end-to-end mode. The paired-end reads were treated as single-end reads. Based on the number of reads mapped onto the unigene sequences, the RPKM (Reads Per Kilobase per Million mapped reads) value of each gene was calculated by an in-house Perl script. We tested for differences between the normalized means of AA and MALs compared with FF as a control. Comparisons were accepted to be significant at adjusted-P value ≤ 0.05 with RPKM fold change ≥ 2 (up-regulated) and ≤ 0.5 (down-regulated) using R v3.2.2 (https://www.r-project.org).

### Saponin pathway analyses

Saponin pathway assignments were carried out using online KEGG mapper (http://www.genome.jp/kegg/tool/map_pathway2.html).

### Submitting RNA-Seq data

The obtained RNA-Seq data from AA, MALs and FF were submitted in DNA Data Bank of Japan (DDBJ) under the following accession numbers:

Submission: DRA005096 (hirakawa-0068_Submission)

BioProject: PRJDB3595 (PSUB004388)

BioSample: SAMD00059523-SAMD00059582 (SSUB006662)

Experiment: DRX062890-DRX062949 (hirakawa-0068_Experiment_0001–0060)

Run: DRR068940-DRR068999 (hirakawa-0068_Run_0001–0060)

## Results

### Genetic effects of the *A*. *cepa* Aggregatum group on the saponin profile of *A*. *fistulosum*

**Fig 1:**
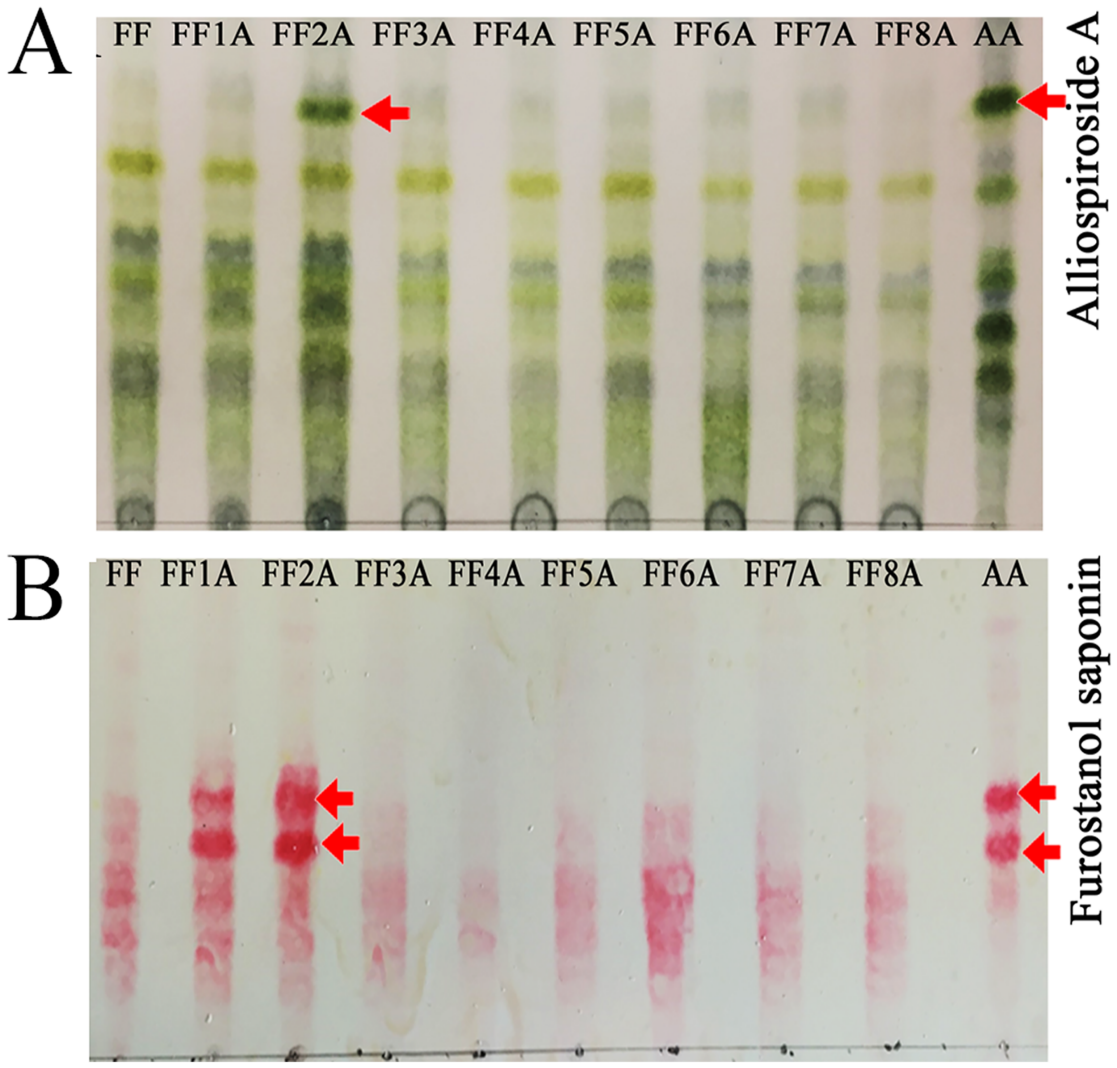
*Allium cepa* Aggregatum group (AA), monosomic addition lines (MALs = FF1A, FF2A, FF3A, FF4A, FF5A, FF6A, FF7A, and FF8A) and *A*. *fistulosum* (FF) saponin TLC profile. (A) Total saponin profile visualized by p-anisaldehyde reagent; arrow indicates the Alliospiroside A accumulation in AA and FF2A. (B) Furostanol saponin profile visualized by Ehrlich's reagent; arrow indicates the furostanol saponin accumulation in AA, FF1A, and FF2A.

**Fig 2:**
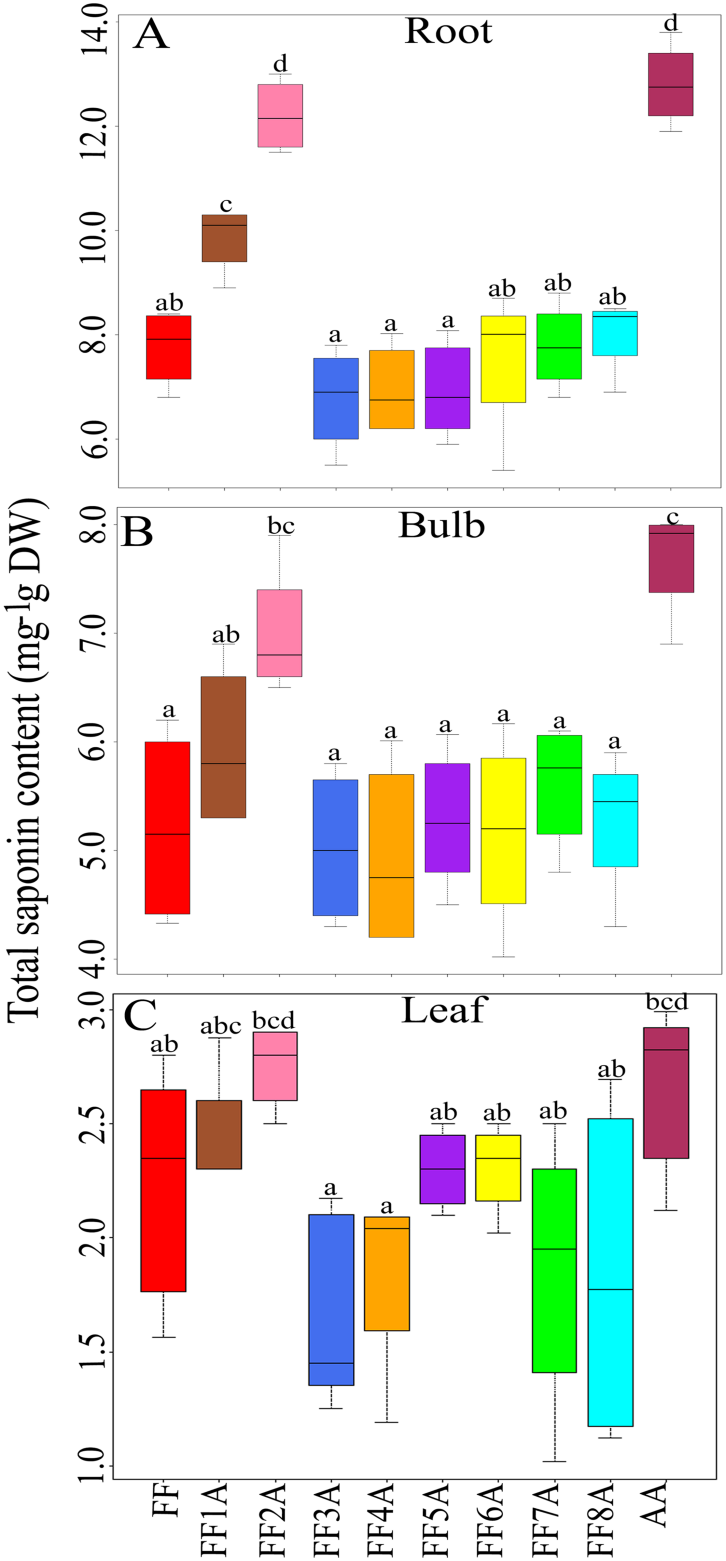
Box plot diagram showing the changes in (A) root, (B) bulb (C) and leaf saponin contents (mg g−1 DW) in *Allium cepa* Aggregatum group (AA), monosomic addition lines (MALs = FF1A, FF2A, FF3A, FF4A, FF5A, FF6A, FF7A, and FF8A) and *A*. *fistulosum* (FF). Values represent the maximum, third quartile, median, first quartile and minimum of three independent replicates (n = 3). Different letters indicate statistically significant difference at *P* < 0.05 according to Tukey’s honest significant difference (HSD) *post-hoc* test.

**Fig 3:**
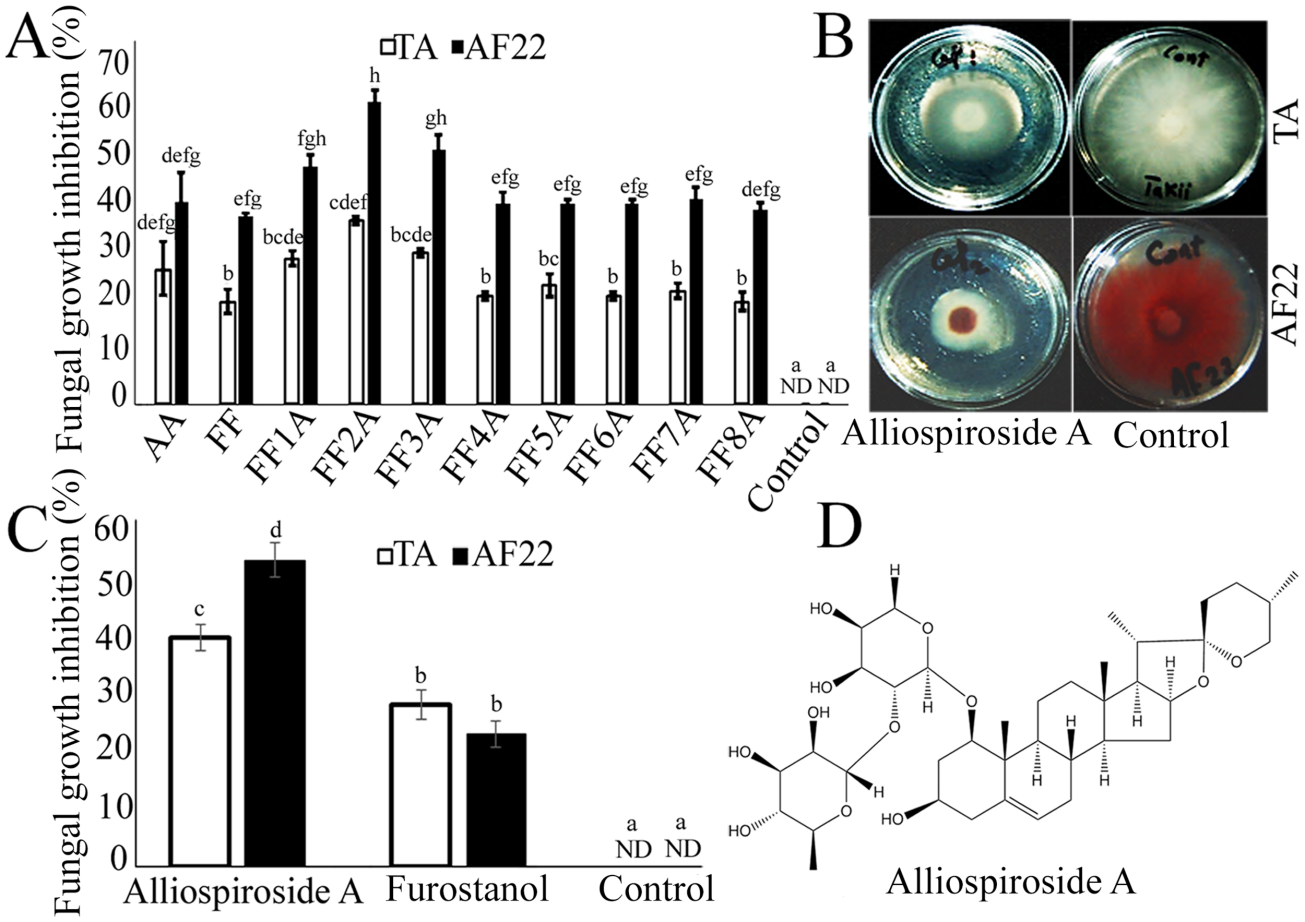
The biological assays of *Allium cepa* Aggregatum group (AA), monosomic addition lines (MALs = FF1A, FF2A, FF3A, FF4A, FF5A, FF6A, FF7A, and FF8A) and *A*. *fistulosum* (FF) crude saponin extracts and Alliospiroside A against *Fusarium oxysporum* f.sp. *cepa*. (A) AA-, MALs- and FF-root saponin extracts antifungal activity against *F*. *oxysporum* f.sp. *cepa* strain TA and AF22. (B) Potato dextrose agar (PDA) plates of Alliospiroside A antifungal activity against *F*. *oxysporum* f.sp. *cepa* strain TA and AF22. (C) Alliospiroside A and furostanol saponin antifungal activity against *F*. *oxysporum* f.sp. *cepa* strain TA and AF22. (D) Alliospiroside A chemical structure. Antifungal activity values are means of three independent replication (*n* = 3) ± standard errors (SEs). Different letters indicate statistically significant differences according to Tukey’s honestly significant difference (HSD) *post-hoc* test.

To understand the functional role of the *A*. *cepa* Aggregatum group (AA) saponin in the improved disease resistance of *A*. *fistulosum* (FF) against *Fusarium* pathogens, the crude saponin extracts from the roots of AA, MALs, and FF were subjected to TLC analyses. The saponin TLC profile revealed a distinctive saponin spot in the AA and FF2A (Fig 1A); however, this saponin compound was missing in other MALs and FF. In addition, two furostanol saponin compounds were clearly accumulated in the AA, FF1A, and FF2A relative to other MALs and FF (Fig 1B). Total saponin contents were highly abundant in the root followed by bulb and leaf tissues (Fig 2), and the highest saponin accumulation was detected in AA, FF1A and FF2A root relative to FF and other MALs root (Fig 2A). Based on the observed results, we hypothesized that a set of saponin biosynthesis-related genes could be allocated in chromosome 2A of the shallot, and these genes are responsible for the distinctive saponin compound biosynthesis. In addition, a set of furostanol saponin biosynthesis-related genes could be also allocated in chromosomes 1A and 2A. To identify this shallot-specific saponin compound in FF2A, crude saponin extract from roots was subjected to column chromatography using a gradient solvent system that yielded a partially purified fraction F2 (18 mg), which was further purified by TLC to obtain 10 mg of the pure compound. The structure of the isolated pure compound was elucidated by 600 MHz NMR analyses (Fig 3D). The ^1^H NMR and ^13^C NMR data of the pure compound was identical to that of Alliospiroside A [35] (S1 Table). The Alliospiroside A is a spirostanol saponin with two glycoside units [[(25S)-3β-hydroxyspirost-5-en-1β-yl] 2-O-(6-deoxy-α-L-mannopyranosyl)-α-L-arabinopyranoside] and a molecular formula of C_38_H_60_O_12_.

To validate the functional role of Alliospiroside A in disease resistance against *Fusarium* pathogens, crude saponin extracts from AA, MALs, and FF roots were examined against *F*. *oxysporum* f. sp. *cepa* strain TA and AF22 using agar diffusion method. The highest significant (*P* ≤ 0.05) fungal growth inhibition percentage was detected in FF2A-root saponin extract with 35.41 and 58.33% against *F*. *oxysporum* f. sp. *cepa* strains TA and AF22, respectively (Fig 3A). In addition, Alliospiroside A antifungal activity was revealed to be 39.58 and 53.12%; however, the furostanol saponin fraction showed 28.12 and 22.91% fungal growth inhibition against the respective strains (Fig 3B and 3C).

### Analysis of differential gene expression in the root, bulb, and leave of AA and MALs as compared with those of FF

The abundance of a transcript in a cDNA library from specific tissues usually corresponds to its expression level, which can indicate the enduring biological processes [36]. In the present study, we accumulated RNA-Seq data from 30 cDNA libraries of root, bulb, and leave of AA, MALs, and FF. The obtained transcriptomic data were deposited in the open access *Allium* Transcript Database (*Allium* TDB, http://alliumtdb.kazusa.or.jp). To identify genes with different expression levels in the root, bulb, and leave of AA and MALs as compared with those of FF (as a control), we initially used the RPKM fold change and false discovery rate (FDR < 0.05) to determine the differential expression. The obtained DGE data are summarized (S2 Table). AM scatter plots of the DGE data of the root, bulb, and leave of AA and MALs versus those of FF (as a control) were carried out (S1–S3 Figs) using average counts and log2 fold changes of RPKM values. The DGE data showed strong up-regulation in AA genotypes as compared with FF at the constative level; 8760, 12354, and 8773 contigs were up-regulated in AA root, bulb, and leave, respectively, and 1910, 1697, and 2321 contigs were up-regulated in FF2A root, bulb, and leave, respectively, as compared with those of FF (S2 Table). The overview of the transcriptome level in each tissue per genotype and its cross-linked expression with AA revealed 1899, 1163, and 1170 contigs were commonly up-regulated in AA and FF2A root, bulb, and leave, respectively; 375, 467, and 413 contigs were commonly down-regulated in AA and FF2A root, bulb, and leave, respectively (S4 Fig).

### Candidate genes involved in the steroidal saponin biosynthesis pathway

**Fig 4:**
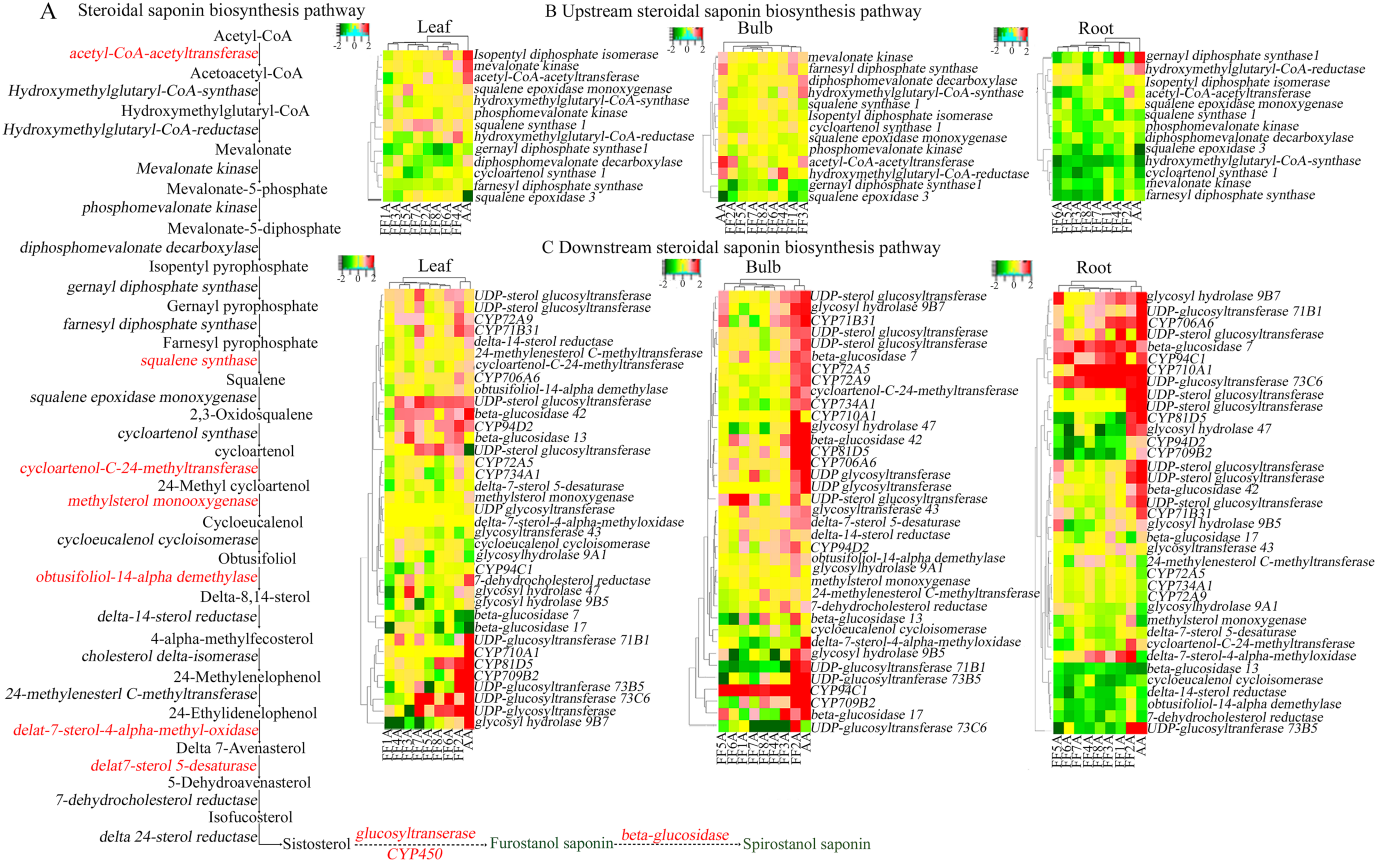
Transcriptomic profiling of steroidal saponin pathway in the root, bulb and leaf of *Allium cepa* Aggregatum group (AA) and monosomic addition lines (MALs = FF1A, FF2A, FF3A, FF4A, FF5A, FF6A, FF7A and FF8A) as compared with *A*. *fistulosum* (FF) as control. (A) Schematic representation of the steroidal saponin biosynthesis pathway and heatmap clustering of the 50 unigene detected in this study which functionally involved in the upstream (B) and downstream (C) saponin biosynthesis pathway. Heatmap constructed using FPKM log2 fold change.

The Kyoto Encyclopedia of Genes and Genomes (KEGG) assignments provide functional annotation of gene-associated biochemical pathways with their corresponding enzyme commission (EC) [37]. Based on similar searches in the KEGG database and our *Allium* unigene sequences, multiple transcript-encoding enzymes involved in the MVA pathway and saponin biosynthesis pathway were identified. We found 50 unigenes in the root, bulb, and leaf of AA and MALs that were functionally involved in saponin biosynthesis (Table 1). In some cases, more than one unique sequence was annotated on the same gene. These unique sequences may represent either different fragments of a single transcript or different parts of a gene or both. Clustering analysis of the unigene dataset based on their expression levels in the AA and MALs revealed a clear clustering of AA and FF2A from other MALs in the saponin downstream pathway (Fig 4A and 4C). *Acetyl-CoA-acetyltransferase* (CL6820.Contig3) and *squalene synthase* (Unigene26049) are two highly expressed saponin-related transcripts in FF2A at the upstream level (Fig 4B). However, most of the up-regulated genes were located at the downstream level, including *cycloartenol-C-24-methyltransferase* (Unigene11966), *methylsterol monooxygenase* (Unigene21326), *Obtusifoliol-14-alpha-demethylase* (Unigene27267), *delta-7-sterol-4-alpha-methyl-oxidase* (Unigene39702), and *delta-7-sterol-5-desaturase* (CL1225.Contig2) (Fig 4C). Further hydroxylation, oxidation and glycosylation steps of the saponin compound via cytochrome P450 and UGT family transcripts, respectively were remarkably up-regulated in the FF2A line (Fig 4C). The final cyclization of the saponin compound via *glucosidase* family transcripts including *beta-glucosidase 7*, *13*, *17*, and *42* (Unigene27678, CL5385.Contig1, Unigene27758, and CL534, respectively), and *glycosyl hydrolase 9A1*, *9B5*, *9B7*, and *47* (*HG9A1*, *GH9B5*, *GH9B7*, and *GH47*, respectively) (CL28.Contig2, CL1599.Contig3, CL1599.Contig2, and Unigene34243, respectively) was also up-regulated in the FF2A line. The transcript data of the downstream saponin biosynthesis pathway revealed up-regulation in the FF2A root and bulb when cross-linked with the AA genotype (Fig 4C).

**Table 1:** List of identified genes involved in the saponin biosynthesis pathway with their unigene. Kyoto Encyclopedia of Genes and Genomes (KEGG) enzyme commission (EC) number and read length (base pair, bp).

Gene name	Unique sequence	KEGG enzyme commission (EC)	Read length (bp)
*acetyl-CoA acetyltransferase*	CL6820.Contig3	[EC:2.3.1.9]	685
*hydroxymethylglutaryl-CoA-synthase*	CL2608.Contig3	[EC:2.3.3.10]	1827
*hydroxy methylglutaryl-CoA-reductase*	Unigene29227	[EC:1.1.1.34]	1926
*mevalonate kinase*	CL3170.Contig2	[EC:2.7.1.36]	1256
*phosphomevalonate kinase*	Unigene11622	[EC:2.7.4.2]	2283
*diphosphomevalonate decarboxylase*	Unigene27463	[EC:4.1.1.33]	1584
*isopentenyl diphosphate isomerase 1*	Unigene3676	[EC:5.3.3.2]	871
*geranyl diphosphate synthase 1*	Unigene37026	[EC:2.5.1.1 2.5.1.10 2.5.1.29]	206
*farnesyl diphosphate synthase*	Unigene27333	[EC:2.5.1.1 2.5.1.10]	1394
*squalene synthase 1*	Unigene26049	[EC:2.5.1.21]	524
*squalene epoxidase monooxygenase 1*	CL5543.Contig2	[EC:1.14.14.17]	1922
*squalene epoxidase 3*	CL5543.Contig3	[EC:1.14.14.17]	302
*cycloartenol synthase 1*	CL2408.Contig1	[EC:5.4.99.8]	2718
*cycloartenol-C-24-methyltransferase*	Unigene11966	[EC:2.1.1.41]	1406
*methylsterol monooxygenase*	Unigene21326	[EC:1.14.13.72]	1259
*cycloeucalenol cycloisomerase*	CL6464.Contig1	[EC:5.5.1.9]	1252
*obtusifoliol (Sterol) 14-alpha demethylase*	Unigene27267	[EC:1.14.13.70]	1826
*delta(14)-sterol reductase*	Unigene28058	[EC:1.3.1.70]	1358
*7-dehydrocholesterol reductase*	Unigene27272	[EC:1.3.1.21]	1669
*delta-7-sterol 4-alpha-methyl-oxidase*	Unigene39702	[EC:1.14.13.72]	152
*24-methylenesterol C-methyltransferase 2*	CL4881.Contig1	[EC:2.1.1.143]	168
*delta-7-sterol-C5(6)-desaturase*	CL1225.Contig2	[EC:1.14.19.20]	639
*UDP-sterol-glucosyltransferase*	Unigene10659	[EC:2.4.1.173]	224
*UDP-sterol-glucosyltransferase*	CL141.Contig10	[EC:2.4.1.173]	1000
*UDP-sterol-glucosyltransferase*	Unigene26213	[EC:2.4.1.173]	736
*glycosyltransferase*, *family 43*	CL1481.Contig2	[EC:2.4.1.17]	1568
*UDP-sterol-glucosyltransferase*	CL141.Contig4	[EC:2.4.1.173]	1657
*UDP-glycosyltransferase*	Unigene24555	[EC:2.4.1.17]	441
*UDP-glycosyltransferase*	Unigene15513	[EC:2.4.1.17]	294
*UDP-glucosyltransferase73B5*	Unigene27343	[EC:2.4.1.17]	1509
*UDP-glucosyltransferase71B1*	CL2556.Contig3	[EC:2.4.1.17]	227
*UDP-glucosyltransferase 73C6*	Unigene18419	[EC:2.4.1.-]	535
*CYP72A5*	CL2624.Contig2	[EC:1.14.-.-]	1430
*CYP709B2*	Unigene21995	[EC:1.14.-.-]	1794
*CYP734A1*	CL2624.Contig3	[EC:1.14.-.-]	1392
*CYP72A9*	CL2624.Contig4	[EC:1.14.-.-]	1177
*CYP94D2*	CL3985.Contig1	[EC:1.14.-.-]	1364
*CYP71B31*	Unigene25910	[EC:1.14.-.-]	430
*CYP81D5*	CL5820.Contig2	[EC:1.14.-.-]	736
*CYP94C1*	Unigene12533	[EC:1.14.-.-]	1867
*CYP706A6*	Unigene416	[EC:1.14.-.-]	1024
*CYP710A1*	Unigene30597	[EC:1.14.19.41]	467
*beta-glucosidase 7*	Unigene27678	[EC:3.2.1.58]	1402
*beta glucosidase 13*	CL5385.Contig1	[EC:3.2.1.21]	193
*beta-glucosidase 17*	Unigene27758	[EC:3.2.1.58]	1674
*beta glucosidase 42*	CL534.Contig1	[EC:3.2.1.21]	841
*glycosyl hydrolase 9A1*	CL28.Contig2	[EC:3.2.1.-]	2243
*glycosyl hydrolase 9B5*	CL1599.Contig3	[EC:3.2.1.-]	282
*glycosyl hydrolase 9B7*	CL1599.Contig2	[EC:3.2.1.-]	744
*glycosyl hydrolase family 47*	Unigene34243	[EC:3.2.1.113]	432

## Discussion

In our previous studies, we were able to illustrate the potential role of crude saponins of shallots for improving the disease resistance of Japanese bunching onion through chromosomal engineering techniques with a certain focus on the phenotypic characters [18]. This study extends the previous work to identify the shallot-specific saponin compound involved in the mechanism of disease resistance as well as candidate genes regulating the steroidal saponin biosynthesis pathway in FF2A using high-throughput RNA-Seq analyses. As far as we know, this is the first study addressing the high-throughput RNA-Seq analysis of saponin pathways in *Allium* species. A more detailed understanding of the saponin compounds and biosynthetic-related genes would facilitate the development of plants with unique saponin contents, either via classical plant breeding or by gene transformation.

Recently, interest in the biosynthesis of steroidal saponins has increased due to their scientific importance in the biomedical application as well as their pivotal role in plant defenses [38, 39]. *Allium* species are rich sources of steroidal saponins, and various saponin compounds isolated from *Allium* species with cytotoxic and antifungal activity have been reported [4, 40, 41]. Previous studies have reported that the initial steps of saponin biosynthesis occur in the leaves, while the later steps of the modification and storage of saponins occur in the roots [19, 42]. Our recent studies have revealed high accumulations of saponin contents in the bulb basal stem and roots of different *Allium* species [3, 4]. The accumulation of saponins in these organs could be related to the physiological role of saponins as a chemical barrier against soil-born fungal pathogens [3, 4, 43]. In the present study, the root saponin TLC profile of AA, MALs, and FF revealed a shallot-specific saponin compound in FF2A (Fig 1A). The observed compound was isolated and identified using column chromatography and 2D NMR spectroscopy. The compound’s identification and structure elucidation were identical to those of spirostanol saponin Alliospiroside A [35] (S1 Table). The saponin extract of FF2A roots revealed significant antifungal activity in comparison with saponin extracts of AA, MALs, and FF roots against *Fusarium* pathogens (Fig 3A). These results indicate the importance of Alliospiroside A as a major bioactive compound against *Fusarium* pathogens and the significant role of spirostanol saponins in disease resistance in comparison with the furostanol type. The obtained results give a better physiological explanation of the phenotypic observation of *Fusarium* basal rot disease resistance in FF2A [18]. Our results were in accordance with a recent report that showed higher fungal growth inhibition of different phytopathogens treated with Alliospiroside A than of those treated with other saponin compounds [35]. The accumulation of Alliospiroside A and two furostanol saponin compounds in the FF2A line was probably due to the saponin genes allocated in chromosome 2A. To validate this hypothesis, a high-throughput RNA-Seq was performed using NGS technology.

RNA-Seq for transcriptome profiling using the NGS technique has shown great potential for functional gene mining and can help in gene discovery, due to its great sequencing depth [44]. *Squalene monooxygenase/epoxidase* catalyzes the conversion of squalene into 2, 3-oxidosqualene acting as a precursor in the biosynthesis of both triterpenes and steroidal saponins in plants [39, 45]. The cyclization of 2, 3-oxidosqualene by the activity of OSC is the branch point for sterol and triterpenoid biosynthesis in many plants [39, 45, 46]. One *OSC* gene, encoding *CAS1*, was detected in our database. The cyclization of 2, 3-oxidosqualene through the activity of CAS leads to the production of cycloartenol and subsequent methylation by *cycloartenol-C-24-methyltransferase* [45, 46, 47, 48]. Steroidal saponins are thought to be derived from cycloartenol formation through the downstream phytosterol pathway. However, the steps at which steroidal saponins and phytosterol biosynthesis diverge have not been clarified, although cholesterol has been suggested as a candidate precursor of steroidal saponins [49, 50, 51]. Our results revealed the up-regulation of *cycloartenol-C-24-methyltransferase* in FF2A bulb and root which could be a candidate gene involved in Alliospiroside A accumulation (Fig 4A and 4C). Additional hydroxylation and oxidation steps are catalyzed by P450 family genes; this step contributes to increasing structural diversity [39; 51, 52]. P450 is one of the largest and most diverse gene families in plants, and only a few P450s have been identified in saponin-involved biosynthesis [39, 53]. In the present study, *CYP51G1* (*obtusifoliol-14-alpha-demethylase*) revealed up-regulation in FF2A bulbs (Fig 4A and 4C). Cytochrome P450 family 51 is essential for sterol and steroidal saponin biosynthesis pathway [27]. *CYP734A1* (CL2624.Contig2) and *CYP72B1* (CL2624.Contig3) were up-regulated in FF2A bulb. These P450s have been reported in brassinosteroid catabolism via C-26 hydroxylation [54]; however, no information has been reported previously about their role in steroidal saponins. In addition, *CYP71B31* was up-regulated in FF2A bulb and leaf, which has been reported in terpenoid biosynthesis [55]. *CYP94C1*, which was involved in Jasmonoyl-isoleucine oxidation [56], was up-regulated in FF2A bulb. The unigene dataset of the P450 family (Table 1) generated in this study provides a significant resource for further molecular and biochemical studies regarding the functional role of these candidates in the biosynthesis of steroidal saponins.

Saponins have one or more sugar chains attached to their aglycone structure through glycoside linkages, and these saccharide moieties can be linear or branched [25]. Sugar moieties are a determining factor in the antifungal activity and hydrophilic properties of saponin compounds [40, 41]. The UGT family catalyzes the transfer of glycosyl residues to precursors that are decorated by P450s. Similarly to the P450, the UGT gene family is large and diverse. Few reports have characterized the UGT family’s role in saponin glycosylation, including the role of *SaGT4A* in the steroidal saponins of *Solanum aculeatissimum* [25], of *UGT71G1* and *UGT73K1* in the triterpenoid saponins of *Medicago truncatula* [57], and of *GmSGT2* in soyasaponins of *Glycine max* [58]. In the present study, *SGT*, *UGT*, *GT43*, *UGT73B5*, *UGT71B1*, and *UGT73C6* were up-regulated in FF2A root and bulb (Fig 4A and 4C). *UGT73C6* was functionally reported in the glycosylation of flavonoids [59], and *UGT73B5* was reported in the hyper-responsive mechanism of *Arabidopsis* against the *Pseudomonas syringae* pv. tomato [60]. The *SGT* family catalyzes the transfer of sugar molecules into diverse sterol molecules and secondary metabolites [61]. Previous studies of the functionality of UGTs revealed a dual nature, and the oligosaccharide extension step in saponin glycosylation is catalyzed by multiple UGTs rather than by a single enzyme [25, 60].

In the present study, the spirostanol saponin Alliospiroside A was shown to play a pivotal role in disease resistance as compared with furostanol saponin (Fig 3C). Similar reports have addressed the importance of spirostanol saponins over the furostanol type [40, 41]. Therefore, increasing spirostanol saponin through classical breeding or gene transformation techniques would be a useful approach for achieving plant resistance against diseases. Beta-glucosidase is the key enzyme that catalyzes the conversion of furostanol saponins into spirostanol by the cleavage of the C-26-bound glucose moiety of furostanol glycosides [62]. Several recent reports have discussed the functional role of beta-glucosidase in the formation of spirostanol saponins, including several spirostanol saponins obtained from seeds of *Trigonella foenum*-*graecum* after the enzymatic hydrolysis of the furostanol saponin fraction by beta-glucosidase [63], and garlic furostanol proto-eruboside-B(1) conversion into spirostanol saponin eruboside-B(2) [64]. In the present study, *beta-glucosidases 7*, *13*, *17*, and *42*, and *GH9A1*, *GH9B5*, *GH9B7*, and *GH47* revealed strong up-regulation in FF2A bulb (Fig 4A and 4C). These candidate genes might be involved in the enzymatic conversion of furostanol saponins into the spirostanol saponin Alliospiroside A in FF2A and its subsequent disease resistance against *Fusarium* pathogens.

## Conclusion

**Fig 5:**
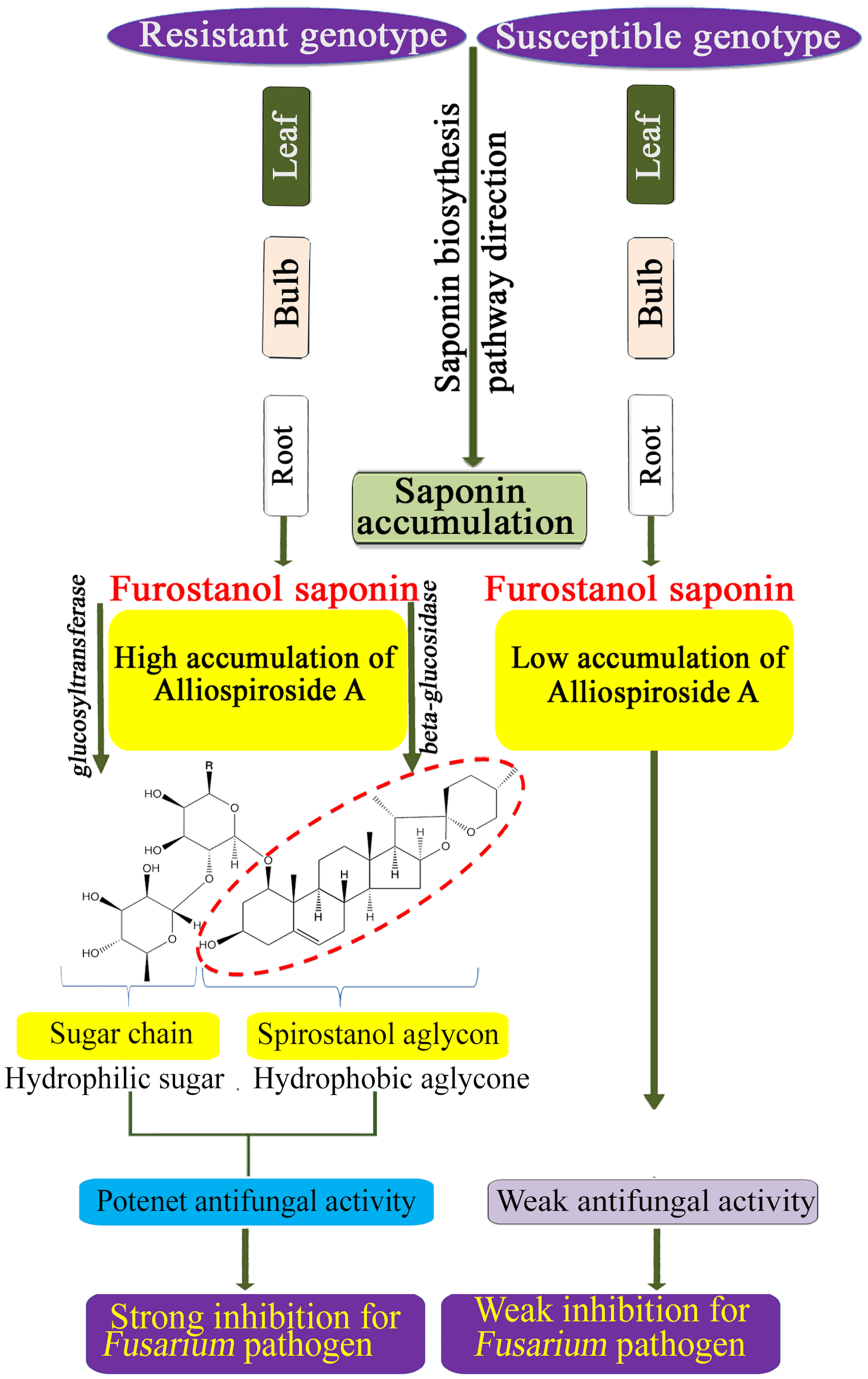
Representative model of Alliospiroside A biosynthesis and defense mechanism in resistance and susceptible *Allium* genotypes against *Fusarium* pathogens.

In this study, phytochemical analyses resulted in the isolation and identification of the spirostanol saponin Alliospiroside A in *A*. *fistulosum* with additional chromosome 2A from shallot (FF2A). Alliospiroside A was the major bioactive compound against *Fusarium* pathogens and a potential chemical marker for disease-resistant genotype selection (Fig 5). The transcriptome of the root, bulb, and leaf of AA, MALs, and FF was obtained using RNA-Seq. The DGE data revealed many up-regulated genes in the biosynthetic pathway of steroidal saponins in the FF2A line. A series of candidate genes involved in the downstream level of the saponin pathway was identified including *Cytochrome P450*, *glycosyltransferases*, and *beta-glucosidase*. Furthermore, clustering analysis of the saponin transcriptional genes revealed a distinctive segregation of the AA and FF2A in the saponin downstream pathway. The obtained results imply the genetic effect of the *A*. *cepa* Aggregatum group on *A*. *fistulosum* saponin physiology and bioactivity. The unigene dataset that was generated in this study provides a significant resource for further molecular and biochemical studies of steroidal saponins.

## Supporting information

S1 Table _13_C NMR spectroscopic data of the aglycone and sugar moieties of the Alliospiroside A isolated from the root of *Allium fistulosum* with extra chromosome 2A from *A*. *cepa* Aggregatum group. (PDF)

S2 Table Total number of up- and down-regulated genes in the root, bulb and leaf of *Allium cepa* Aggregatum group (AA) and Monosomic Addition Lines (MALs = FF1A, FF2A, FF3A, FF4A, FF5A, FF6A, FF7A and FF8A) in compare to *A*. *fistulosum* (FF) as control. (PDF)

S1 Fig AM scatter plots of the root differential gene expression of *Allium cepa* Aggregatum group (AA) and monosomic addition lines (MALs = FF1A, FF2A, FF3A, FF4A, FF5A, FF6A, FF7A, and FF8A) in comparison with *A*. *fistulosum* (FF) as control. Log2 fold change of AA/FF and MALs/FF on the y-axis and average count of RPKM (Reads Per Kilobase of exon per Million mapped reads) values on the x-axis. Up-regulated genes (Red, fold change > 2 and adjusted-P < 0.05), down-regulated genes (green, fold change < 0.5 and adjusted-P < 0.05), and differential expressed genes (Blue, adjusted-P < 0.05). Non differential expressed genes (black).
(PDF)

S2 Fig AM scatter plots of the bulb differential gene expression of *Allium cepa* Aggregatum group (AA) and monosomic addition lines (MALs = FF1A, FF2A, FF3A, FF4A, FF5A, FF6A, FF7A, and FF8A) in comparison with *A*. *fistulosum* (FF) as control. Log2 fold change of AA/FF and MALs/FF on the y-axis and average count of RPKM (Reads Per Kilobase of exon per Million mapped reads) values on the x-axis. Up-regulated genes (Red, fold change > 2 and adjusted-P < 0.05), down-regulated genes (green, fold change < 0.5 and adjusted-P < 0.05), and differential expressed genes (Blue, adjusted-P < 0.05). Non differential expressed genes (black).
(PDF)

S3 Fig AM scatter plots of the leaf differential gene expression of Allium cepa Aggregatum group (AA) and monosomic addition lines (MALs = FF1A, FF2A, FF3A, FF4A, FF5A, FF6A, FF7A, and FF8A) in comparison with A. fistulosum (FF) as control. Log2 fold change of AA/FF and MALs/FF on the y-axis and average count of RPKM (Reads Per Kilobase of exon per Million mapped reads) values on the x-axis. Up-regulated genes (Red, fold change > 2 and adjusted-P < 0.05), down-regulated genes (green, fold change < 0.5 and adjusted-P < 0.05), and differential expressed genes (Blue, adjusted-P < 0.05). Non differential expressed genes (black).
(PDF)

S4 Fig Venn diagram showing the distribution of common up-regulated and down-regulated genes of *Allium cepa* Aggregatum group (AA) and monosomic addition lines (MALs = FF1A, FF2A, FF3A, FF4A, FF5A, FF6A, FF7A and FF8A) in the Leaf (A), bulb (B) and root (C). (PDF)
