# Correction: RNA-sequencing-based transcriptome and biochemical analyses of steroidal saponin pathway in a complete set of *Allium fistulosum*—*A*. *cepa* monosomic addition lines

**DOI:** 10.1371/journal.pone.0190813

**Published:** 2018-01-02

**Authors:** Mostafa Abdelrahman, Magdi El-Sayed, Shusei Sato, Hideki Hirakawa, Shin-ichi Ito, Keisuke Tanaka, Yoko Mine, Nobuo Sugiyama, Yutaka Suzuki, Naoki Yamauchi, Masayoshi Shigyo

The ninth author's name is incorrect. The correct name is: Yutaka Suzuki.

In the Funding section, the first grant number from JSPS KAKENHI is listed incorrectly. The correct grant number is: 26292020.

There are errors in the author affiliations. The affiliations should appear as shown here:

Mostafa Abdelrahman^1,2,3^, Magdi El-Sayed^2^, Shusei Sato^3^, Hideki Hirakawa^4^, Shin-ichi Ito^1^, Keisuke Tanaka^5^, Yoko Mine^6^, Nobuo Sugiyama^6^, Yutaka Suzuki^7^, Naoki Yamauchi^1^, Masayoshi Shigyo^1^

**1** College of Agriculture, Graduate School of Sciences and Technology for Innovation, Yamaguchi University, Yamaguchi-City, Yamaguchi, Japan, **2** Botany Department, Faculty of Science, Aswan University, Aswan, Egypt, **3** Department of Environmental Life Sciences, Graduate School of Life Sciences, Tohoku University, Sendai-City, Miyagi, Japan, **4** Kazusa DNA Research Institute, Kisarazu-City, Chiba, Japan, **5** NODAI Genome Research Center, Tokyo University of Agriculture, Setagaya-Ku, Tokyo, Japan, **6** Department of Agriculture, Faculty of Agriculture, Tokyo University of Agriculture, Atsugi-City, Kanagawa, Japan, **7** Department of Computational Biology and Medical Sciences, Graduate School of Frontier Sciences, The University of Tokyo, Kashiwa-City, Chiba, Japan
