# Supplementary material for: RNA-sequencing-based transcriptome and biochemical analyses of steroidal saponin pathway in a complete set of Allium fistulosum—A. cepa monosomic addition lines
Source: PLoS One. 2017 Aug 11;12(8):e0181784. doi: 10.1371/journal.pone.0181784 (PMC5553718; doi:10.1371/journal.pone.0181784)
Supplement: S1 Table — (PDF) [file pone.0181784.s001.pdf]

S1 Table. ^{13}C NMR spectroscopic data of the aglycone and sugar moieties of the Alliospiroside A isolated from the root of *Allium fistulosum* with extra chromosome 2A from *A. cepa* Aggregatum group.

**Position**	**Alliospiroside A Chemical shift**	**Position**	**Chemical shift**
C-1	83.65	L-Arabinose-1	100.43
C-2	37.54	2	75.26
C-3	68.28	3	75.90
C-4	43.91	4	70.10
C-5	139.64	5	67.35
C-6	124.76	L-Rhamnose-1	101.70
C-7	33.22	2	72.67
C-8	32.08	3	72.58
C-9	50.49	4	74.30
C-10	42.98	5	69.46
C-11	24.12	6	19.04
C-12	40.42		
C-13	40.25		
C-14	56.90		
C-15	32.46		
C-16	81.28		
C-17	62.91		
C-18	16.78		
C-19	14.91		
C-20	42.52		
C-21	15.10		
C-22	109.78		
C-23	26.47		
C-24	26.26		
C-25	27.61		
C-26	65.11		
C-27	16.26		
