# Supplementary material for: RNA-sequencing-based transcriptome and biochemical analyses of steroidal saponin pathway in a complete set of Allium fistulosum—A. cepa monosomic addition lines
Source: PLoS One. 2017 Aug 11;12(8):e0181784. doi: 10.1371/journal.pone.0181784 (PMC5553718; doi:10.1371/journal.pone.0181784)
Supplement: S2 Table — (PDF) [file pone.0181784.s002.pdf]

S2 Table. Total number of up- and down-regulated genes in the root, bulb and leaf of *Allium cepa* Aggregatum group (AA) and Monosomic Addition Lines (MALs = FF1A, FF2A, FF3A, FF4A, FF5A, FF6A, FF7A and FF8A) in compare to *A. fistulosum* (FF) as control.

		Leaf	
Genotype	Up-regulated	Down-regulated	Differential gene expression
AA	8773	3360	12133
FF1A	1423	1589	3012
FF2A	2321	1565	3886
FF3A	2921	749	3670
FF4A	3692	675	4367
FF5A	1850	1303	3153
FF6A	2645	738	3383
FF7A	3853	793	4646
FF8A	2110	1852	3962
		Bulb	
Genotype	Up-regulated	Down-regulated	Differentially gene expression
AA	12354	1813	14167
FF1A	1350	2350	3700
FF2A	1697	4551	6248
FF3A	2284	1456	3740
FF4A	2062	1483	3545
FF5A	1872	981	2853
FF6A	1975	891	2866
FF7A	1665	789	2454
FF8A	1268	972	2240
		Root	
Genotype	Up-regulated	Down-regulated	Differentially gene expression
AA	8760	4750	13510
FF1A	2213	2424	4637
FF2A	1910	1622	3532
FF3A	2419	4444	6863
FF4A	1769	1291	3060
FF5A	2208	3215	5423
FF6A	2064	4889	6953
FF7A	1665	1843	3508
