# Supplementary material for: RNA-sequencing-based transcriptome and biochemical analyses of steroidal saponin pathway in a complete set of Allium fistulosum—A. cepa monosomic addition lines
Source: PLoS One. 2017 Aug 11;12(8):e0181784. doi: 10.1371/journal.pone.0181784 (PMC5553718; doi:10.1371/journal.pone.0181784)
Supplement: S3 Fig — Up-regulated genes (Red, fold change > 2 and adjusted-P < 0.05), down-regulated genes (green, fold change < 0.5 and adjusted-P < 0.05), and differential expressed genes (Blue, adjusted-P < 0.05). Non differential expressed genes (black). (PDF) [file pone.0181784.s005.pdf]

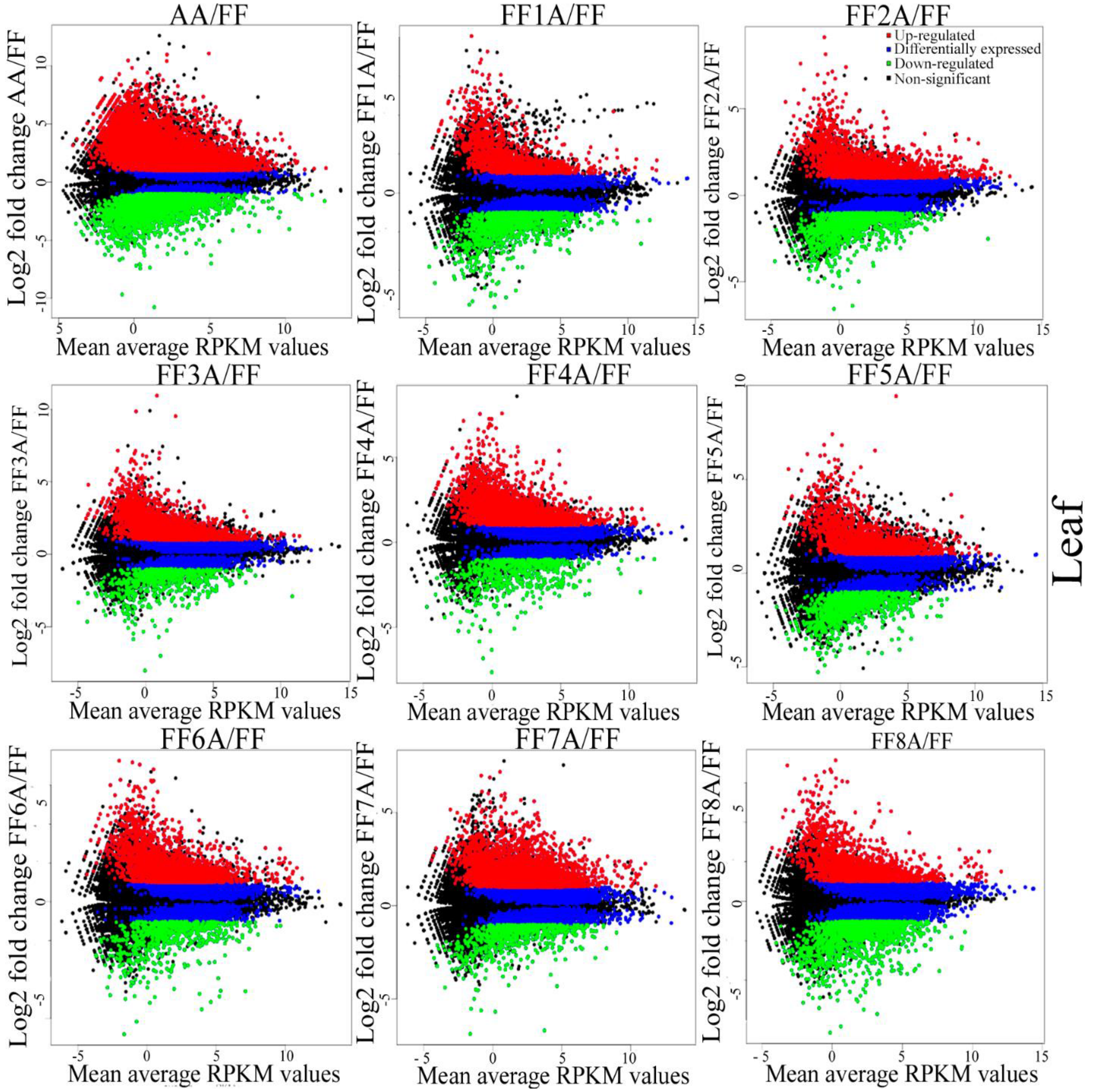

S3 Fig. AM scatter plots of the leaf differential gene expression of Allium cepa Aggregatum group (AA) and monosomic addition lines (MALs = FF1A, FF2A, FF3A, FF4A, FF5A, FF6A, FF7A, and FF8A) in comparison with A. fistulosum (FF) as control. Log2 fold change of AA/FF and MALs/FF on the y-axis and average count of RPKM (Reads Per Kilobase of exon per Million mapped reads) values on the x-axis. Up-regulated genes (Red, fold change > 2 and adjusted-P < 0.05), down-regulated genes (green, fold change < 0.5 and adjusted-P < 0.05), and differential expressed genes (Blue, adjusted-P < 0.05). Non differential expressed genes (black).
