# Supplementary figures and images for: RNA-sequencing-based transcriptome and biochemical analyses of steroidal saponin pathway in a complete set of Allium fistulosum—A. cepa monosomic addition lines
Source: PLoS One. 2017 Aug 11;12(8):e0181784. doi: 10.1371/journal.pone.0181784 (PMC5553718; doi:10.1371/journal.pone.0181784)

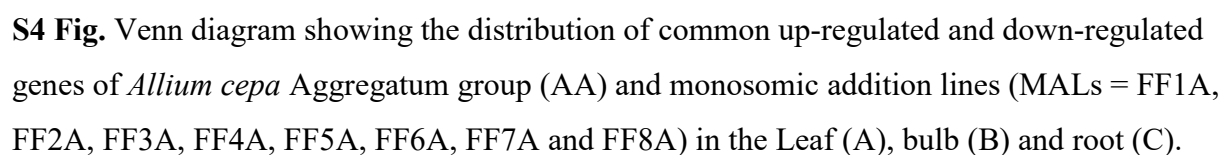

Supplement: S4 Fig — (PDF) [file pone.0181784.s006.pdf]
